# *Ex vivo* chemosensitivity testing and gene expression profiling predict response towards adjuvant gemcitabine treatment in pancreatic cancer

**DOI:** 10.1038/sj.bjc.6604528

**Published:** 2008-08-26

**Authors:** C W Michalski, M Erkan, D Sauliunaite, T Giese, R Stratmann, C Sartori, N A Giese, H Friess, J Kleeff

**Affiliations:** 1 Department of General Surgery, Technische Universität München, Munich, Germany; 2 Institute of Immunology, University of Heidelberg, Heidelberg, Germany; 3 DCS Innov. Diagnostik-Systeme GmbH & Co KG, Hamburg, Germany; 4 Department of General Surgery, University of Heidelberg, Heidelberg, Germany

**Keywords:** chemosensitivity testing, pancreatic cancer, gemcitabine, nucleoside transporter

## Abstract

Efficacy of chemotherapy for pancreatic cancer may be improved by tailoring it to individual chemosensitivity profiles. Identification of nonresponders before initiation of treatment may help to avoid side effects. In this study, primary pancreatic cancer cells were isolated from 18 patients undergoing pancreaticoduodenectomy for pancreatic cancer. Eight commonly used pancreatic cancer cell lines were used as controls. *Ex vivo* chemosensitivity for gemcitabine, 5-fluorouracil, mitomycin-C, cisplatinum, oxaliplatinum, paclitaxel and a combination of gemcitabine with oxaliplatinum or mitomycin-C was determined using a cellular ATP-based tumour chemosensitivity assay (ATP-TCA). Quantitative real-time–polymerase chain reaction was performed to determine RNA expression levels of genes implicated in chemoresistance. Chemosensitivity towards cytotoxic agents was highly variable in primary pancreatic cancer cells and pancreatic cancer cell lines. ATP-TCA results for gemcitabine correlated to the tissue expression of human equilibrative nucleoside transporter-1 (hENT1). Time to relapse in patients with gemcitabine-sensitive tumours was significantly higher than in patients with chemoresistant pancreatic cancers (*P*=0.01; 71 *vs* 269 days). Furthermore, time to relapse in gemcitabine-treated patients was related to hENT1 expression (*P*=0.0067). Thus, chemosensitivity testing using ATP-TCA in pancreatic cancer is feasible and correlated with time to relapse in gemcitabine-treated patients. This suggests that ATP-TCA testing could be used as a decision-making tool in the adjuvant treatment of pancreatic cancer.

Chemotherapy for pancreatic cancer is only partially effective in the adjuvant setting (Neoptolemos *et al*, 2004; Stocken *et al*, 2005; Oettle *et al*, 2007). A large number of patients are resistant to the currently used standard chemotherapeutic regimens, and the benefit of chemotherapy is limited to a small number of patients. Nonetheless, costs and side effects affect all patients treated. The overall prognosis is still dismal with 5-year survival rates not exceeding 4% (Jemal *et al*, 2007). Poor response to chemotherapy may be a result of treating patients without exact knowledge of the underlying molecular and genetic mechanisms of the disease (Damaraju *et al*, 2003; Kornmann *et al*, 2003; Lee *et al*, 2005).

Chemoresistance of tumour cells can be provoked by mutations in oncogenes (eg, K-ras), loss of tumour suppressors (eg, p53, p16^INK4^) or dysregulation of genes involved in cell cycle cell proliferation, signal transduction, angiogenesis or apoptosis (Lee *et al*, 2005; Kleeff *et al*, 2006). A number of studies have analysed genes, which are directly involved in the metabolism of cytotoxic drugs. Although the metabolisation pathways of the chemotherapeutic agents currently used in standard adjuvant treatment of pancreatic cancer (gemcitabine, 5-fluorouracil and platinum compounds) are complex, some genes that alter uptake, metabolisation and catabolisation of these substances have been shown to be associated with chemoresistance.

The first step of gemcitabine metabolism is the uptake into the cell, which is accomplished by the human nucleoside equilibrative transporter 1 (hENT1) (Damaraju *et al*, 2003; Spratlin *et al*, 2004; Nakano *et al*, 2007; Giovannetti *et al*, 2006a, 2006b). Conversion of gemcitabine prodrug to the active diphosphate and triphosphate metabolites is performed by deoxycytidine kinase (DCK) (Sebastiani *et al*, 2006), whereas catabolism of gemcitabine phosphates is carried out by the enzymes 5′-nucleotidase and cytidine deaminase (CDA) (Maring *et al*, 2005). Dihydropyrimidine dehydrogenase (DPYD) (Nakayama *et al*, 2004; Giovannetti *et al*, 2006a, 2006b), thymidine synthase and thymidine phosphorylase (TP) (Passantino *et al*, 2005) have been shown to be key enzymes in the metabolism of 5-fluorouracil and also in the development of chemoresistance to this drug.

Although numerous studies have addressed chemosensitivity testing in a variety of malignancies, there are only a few studies in pancreatic cancer. As standard chemotherapy has only limited effects in the palliative and in the adjuvant setting, it is of importance to identify those patients who will benefit from a specific chemotherapy. Although *in vitro* chemosensitivity testing is a very attractive concept at first glance, it might not necessarily predict *in vivo* responses due to a number of different factors. Sub-populations of tumour cells may expand clonally in cell culture, and the absence of immune cells and – particularly important – the absence of a functional tumour microenvironment (ie, extracellular matrix, blood vessels, nerves) may influence the clinical response to a given chemotherapy (Miyamoto *et al*, 2004). However, different approaches have been used to predict *ex vivo* chemosensitivity of tumour cells to particular cytotoxic agents, such as profiling of the expression of individual genes implicated in chemoresistance (Erkan *et al*, 2005; Nakahira *et al*, 2007; Nakano *et al*, 2007), high-throughput analysis of many genes (Kornmann *et al*, 2003; Potti *et al*, 2006; Chen *et al*, 2007) and chemosensitivity assays using primary isolated cancer cells.

For *in vitro* chemosensitivity testing of cells, different assays have been developed over time. These can roughly be divided into two groups: clonogenic and non-clonogenic assays. Clonogenic assays first expand tumour cells before treatment with cytotoxic agents. In non-clonogenic assays, primary tumour cells are exposed to chemotherapeutic drugs. The readout of the assays might be measurement of cell proliferation, cell death or metabolic activity. Various types of assays have been tested in a variety of malignancies, but none so far has been evaluated in large-scale randomised clinical trials. In this study, we used the ATP-based tumour chemosensitivity assay (ATP-TCA), which has been shown to be very sensitive and highly reproducible, at a low rate of failure (Andreotti *et al*, 1995; Ugurel *et al*, 2006).

In palliative or neoadjuvant settings, the amount of cancer tissue that can be obtained by endoscopic retrograde cholangiopancreatography and fine needle aspiration is limited, which might preclude the ATP-TCA from being used as a test system. Alternatively, QRT–PCR (quantitative real-time polymerase chain reaction)-driven gene expression analysis (for which the amount of tissue needed for mRNA isolation is rather small) might be more suitable for predicting chemosensitivity in inoperable or neoadjuvant-treated patients.

Thus in our study, two different *in vitro* techniques – ATP-TCA and gene expression profiling – were used to analyse chemosensitivity in eighteen primary human pancreatic cancer cells and eight established pancreatic cancer cell lines. The ATP-TCA included analysis of response of primary tumour cells and established cell lines to gemcitabine and 5-fluorouracil. In addition, we analysed expression of genes implicated in gemcitabine chemoresistance (hENT1, CDA, DCK) and 5-fluorouracil chemoresistance (DPYD, TP).

## Patients and methods

### Patients and tissue sampling

Tissue samples were collected from patients undergoing pancreatic resections for pancreatic cancer (*n*=24). Primary cancer cell isolation was technically insufficient in six patients (too few cancer cells, bacterial contamination). Thus, 18 patients were eligible for inclusion in our study. Table 1 shows the characteristics of the included patients. Resected pancreatic cancer tissues were immediately transferred to culture medium, which had been precooled to 4°C. Chemosensitivity testing was performed within 48 h of sample collection. For gene expression analyses, pancreatic tissues were immediately snap frozen at −80°C and were subsequently processed for mRNA isolation. As a control group for gene expression analyses, normal pancreatic tissue samples were obtained through an organ donor procurement programme whenever there was no suitable recipient for pancreas transplantation (*n*=6). The use of human tissue for the analysis was approved by the local ethical committee (University of Heidelberg, Germany) and written informed consent was obtained from the patients.

### Chemosensitivity testing using the ATP-TCA test

Primary human pancreatic cancer cells were isolated and ATP-TCA was performed as described previously (Andreotti *et al*, 1995; Ugurel *et al*, 2006). Briefly, 10 000–20 000 tumour cells per well were seeded in tumour cell-supporting growth medium in 96-well microtiter plates. Chemotherapeutics were used at test drug concentrations (TDCs) of 200, 100, 50, 25, 12.5 and 6.25% (with 100% TDC corresponding to peak plasma concentrations). The following chemotherapeutic agents or combinations were chosen: 5-fluorouracil, gemcitabine, oxaliplatinum, cisplatinum, paclitaxel, mitomycin C, gemcitabine+oxaliplatinum and gemcitabine+mitomycin C. Tests were performed in triplicate. After 7 days of incubation, tumour cells were lysed and the amount of cellular ATP – which correlates directly with the number of viable cells – was determined with a luciferin/luciferase reaction. Cell preparation and ATP-TCA were performed using commercially available reagents (TCA-100, DCS, Hamburg, Germany). Luminescence was measured with either a LB953 luminometer (Berthold Technologies, Bad Wildbad, Germany) or a MPL2 Microplate Luminometer (Berthold Detection Systems, Pforzheim, Germany). The above-mentioned cytotoxic drugs have been used in the ATP-TCA test in different ‘test drug concentrations’ (TDCs) of which 100% correspond to the peak plasma concentration. The IC_50_ (inhibitory concentration of 50%) is the concentration at which cell growth is inhibited by 50% (which, in the ATP-TCA test, is a combination of cell growth and cell survival); this value is calculated (as described in the ATP-TCA test protocols as supplied by DCS diagnostics, Hamburg, Germany) using an interpolation of two neighbouring measurements. Test values below 50% are considered ‘sensitive’, whereas test values above 100% are considered ‘resistant’.

### Quantitative RT–PCR

Messenger-RNA isolation, cDNA preparation and QRT–PCR were performed as described previously (Michalski *et al*, 2007). Primers detecting hENT1, CDA, DCK, DPYD and TP were obtained from Search-LC (Heidelberg, Germany). The number of specific transcripts detected was normalised to the level of the housekeeping gene cyclophilin-B (CPB) and expressed as the number of target transcripts per 10 000 CPB copies.

### Statistical analysis

Differences in mRNA expression levels of hENT1, CDA, DCK, DPYD and TP were compared using a Mann–Whitney *U*-test. For correlation analysis, the Spearman rho (for primary isolated cancer cell correlations) and Pearson (for pancreatic cancer cell line correlations) tests were used. The survival curve of all patients and the time to relapse/time to progress of gemcitabine-treated patients were presented using the Kaplan–Meier method. Time to relapse of gemcitabine-treated patients with low or high chemosensitivity for gemcitabine was compared using a log-rank test. The level of statistical significance was set at *P*<0.05.

## Results

**Figure 1:**
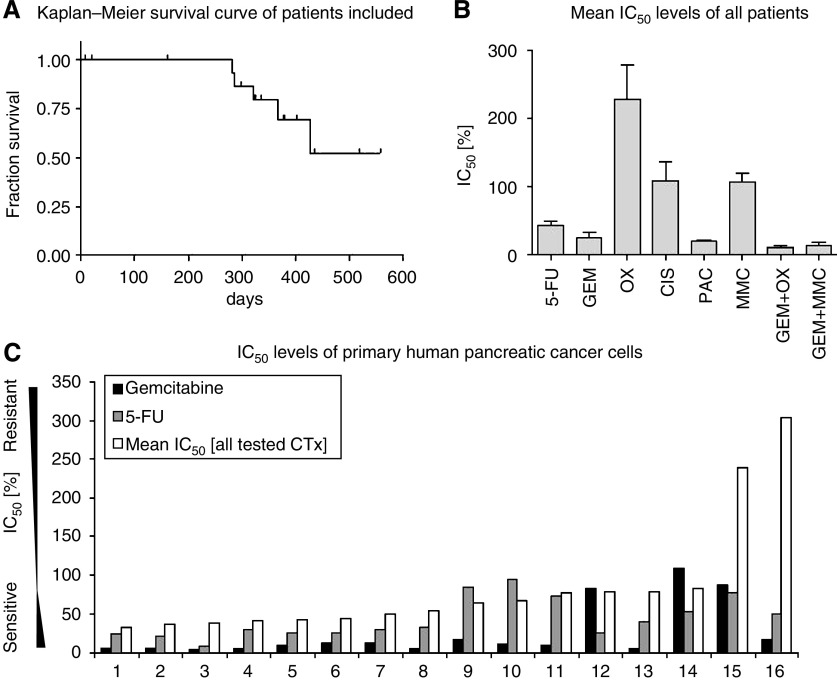
(**A**) Kaplan–Meier survival curve of patients tested with ATP-TCA chemosensitivity assays. (**B**) Mean IC_50_ levels (% of peak plasma concentration) of all patients in this cohort. (**C**) Comparison of the IC_50_ levels of gemcitabine (black bars) and 5-fluorouracil (grey bars) in single patients as compared with the mean IC_50_ levels of all chemotherapeutic drugs (white bars).

Eighteen patients with resectable pancreatic adenocarcinoma were included in the study from September 2005 to January 2006 (Table 1). At the last follow-up (March 2007), five patients had died and thirteen patients were alive. Figure 1A shows the Kaplan–Meier survival curve of this patient cohort. Seven patients received first-line adjuvant chemotherapy with gemcitabine, two patients were included into the CapRI study (for protocol, see reference Knaebel *et al*, 2005), one patient received gemcitabine/capecitabine and eight patients received no chemotherapy. Analysis of seven patients treated with first-line gemcitabine revealed a time to relapse of 193 days, as judged by time from initiation of chemotherapy until radiologically detected recurrence of the disease.

### Chemosensitivity of primary human pancreatic cancer cells

To assess chemosensitivity, the ATP-TCA test was performed on primary (isolated) human pancreatic cancer cells of the 18 included patients. This analysis revealed a large variability in sensitivity to the tested cytotoxic drugs (Figure 1B). Cancer cells were resistant to platinum derivatives and mitomycin C, whereas chemosensitivity was seen for 5-fluorouracil, gemcitabine and paclitaxel (Figure 1B). Chemosensitivity of these cells towards gemcitabine was increased by combining it with either oxaliplatinum or mitomycin C. As examples of the highly variable chemosensitivity, IC_50_ values of 16 patients for gemcitabine and 5-fluorouracil (black and grey bars, respectively; Figure 1C; two patients were excluded due to contamination of the primary isolated cancer cells) are shown in comparison with the mean IC_50_ values of all tested cytotoxic drugs (white bars; Figure 1C).

### Chemosensitivity of pancreatic cancer cell lines

**Figure 2:**
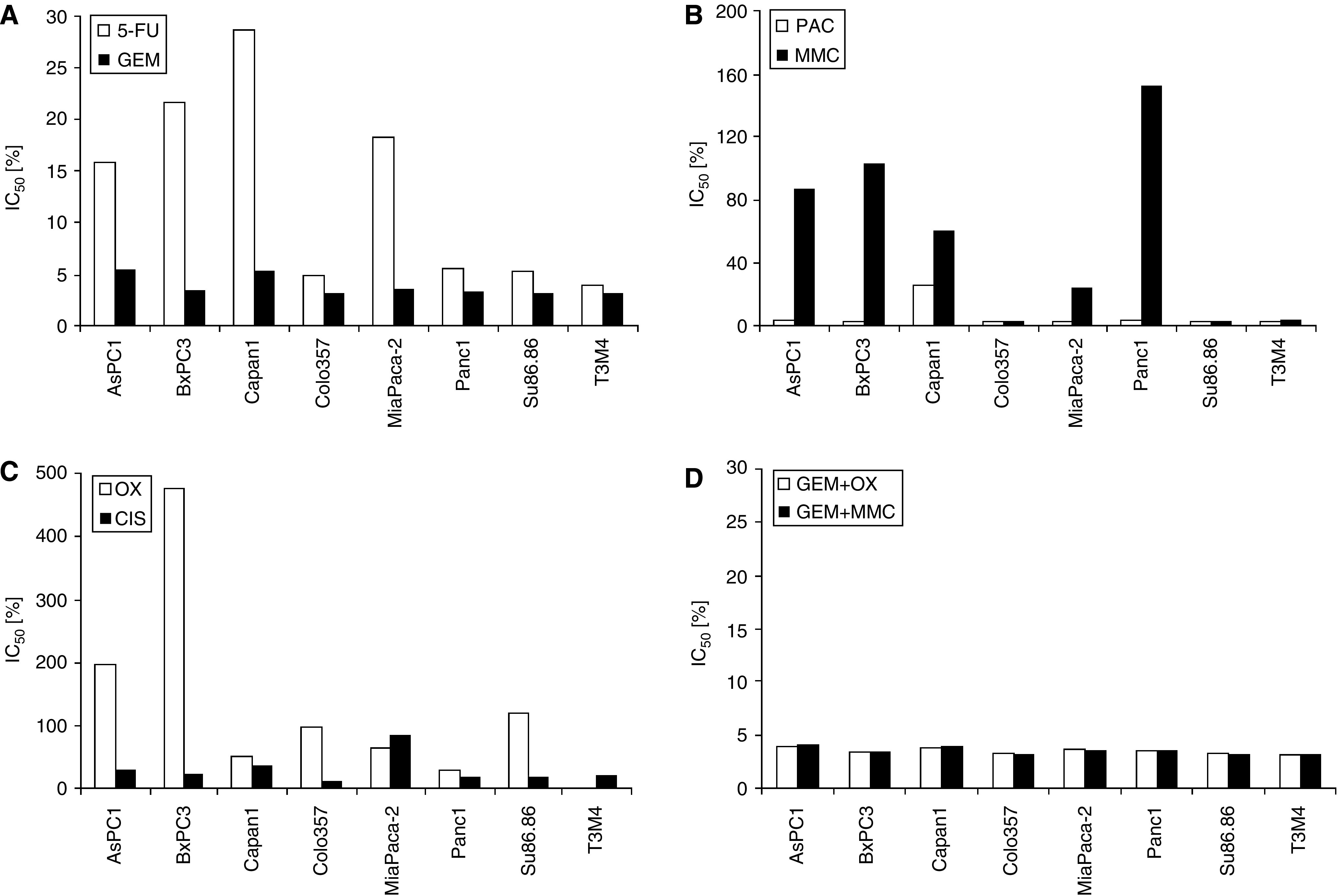
IC_50_ values (% of peak plasma concentration) of eight pancreatic cancer cell lines for (**A**) 5-fluorouracil and gemcitabine (5-FU, GEM; white and black bars; (**B**), paclitaxel and mitomycin-C (PAC, MMC; white and black bars); (**C**), oxaliplatinum and cisplatinum (OX, CIS; white and black bars; (**D**) and for gemcitabine+oxaliplatinum and gemcitabine+mitomycin-C (GEM+OX, GEM+MMC; white and black bars).

**Figure 3:**
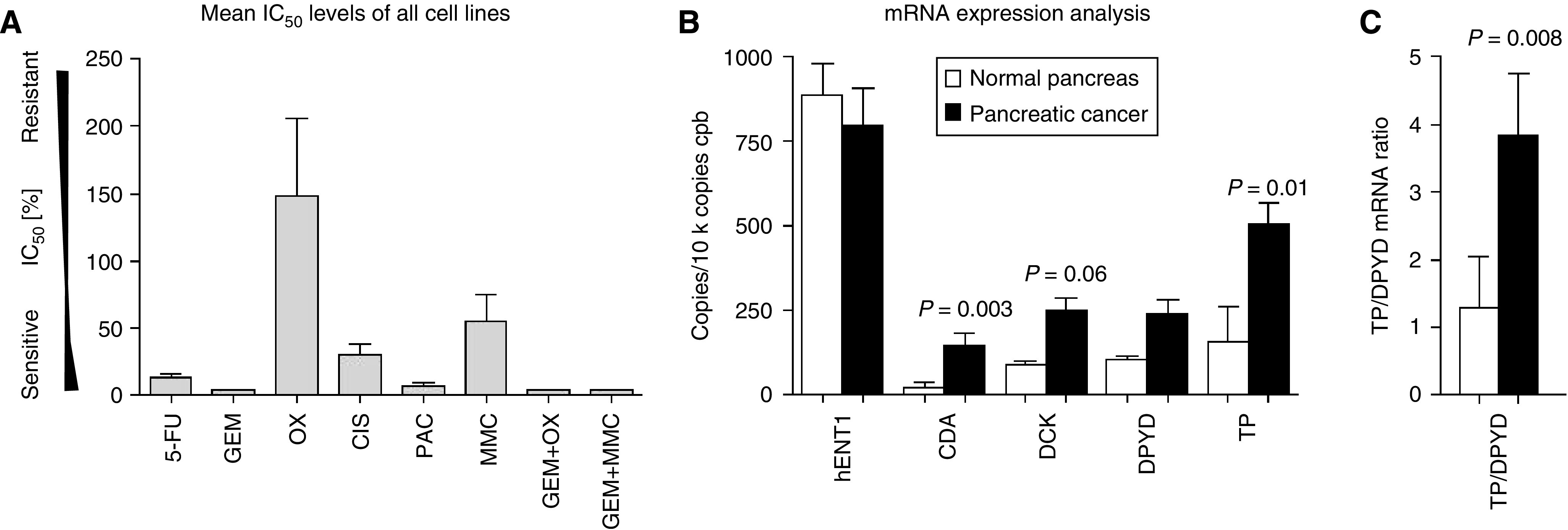
(**A**) Mean IC_50_ levels (% of peak plasma concentration) of eight pancreatic cancer cell lines (AsPC, BxPC3, Capan1, Col357, MiaPaca-2, Panc1, Su86.86, T3M4). (**B**) Expression analysis (quantitative real-time polymerase chain reaction) of genes implicated in chemoresistance (white bars: donor pancreas; black bars: pancreatic cancer; Mann–Whitney *U*-test). (**C**) Thymidine phosphorylase (TP)/ dihydropyrimidine dehydrogenase (DPYD) ratio, normal pancreas *vs* pancreatic cancer.

A similar pattern of chemosensitivity was seen in eight standard pancreatic cancer cell lines tested: AsPC1, BxPC3, Capan-1, Colo357, MiaPaca-2, Panc1, Su86.86 and T3M4 (Figure 2A-D and Figure 3A). Although these were mainly resistant towards oxaliplatinum, cisplatinum and mitomycin C, chemosensitivity was observed for 5-fluorouracil, gemcitabine and paclitaxel. Again, combination therapies with gemcitabine plus oxaliplatinum or mitomycin C rendered the cells even more sensitive than with gemcitabine alone. However, the chemosensitivity profiles varied considerably within these tested cell lines (Figure 2A–D). As judged by the mean IC_50_ levels of all tested cytotoxic drugs, the ‘average’ pancreatic cancer cell line was chemosensitive to 5-fluorouracil, gemcitabine, paclitaxel and gemcitabine combinations, whereas it showed intermediate resistance to cisplatinum and resistance to oxaliplatinum and mitomycin C (Figure 3A).

### Gene expression analysis in primary human pancreatic cancer cells

To analyse potential associations between mRNA expression levels of genes, which are implicated in chemoresistance, QRT–PCR was performed using tissue specimens tested with ATP-TCA and normal pancreas tissues as controls. This analysis (Figure 3B) revealed an increase of CDA (*P*=0.003), DCK (*P*=0.06), DPYD (*P*=ns) and TP (*P*=0.01) in pancreatic cancer tissues, whereas hENT1 (*P*=ns) expression was slightly reduced compared with the normal pancreas. The TP/DPYD ratio – a marker of 5-fluorouracil chemoresistance – was increased in primary pancreatic cancer tissues (*P*=0.008, Figure 3C).

### Gene expression analysis in pancreatic cancer cell lines

**Figure 4:**
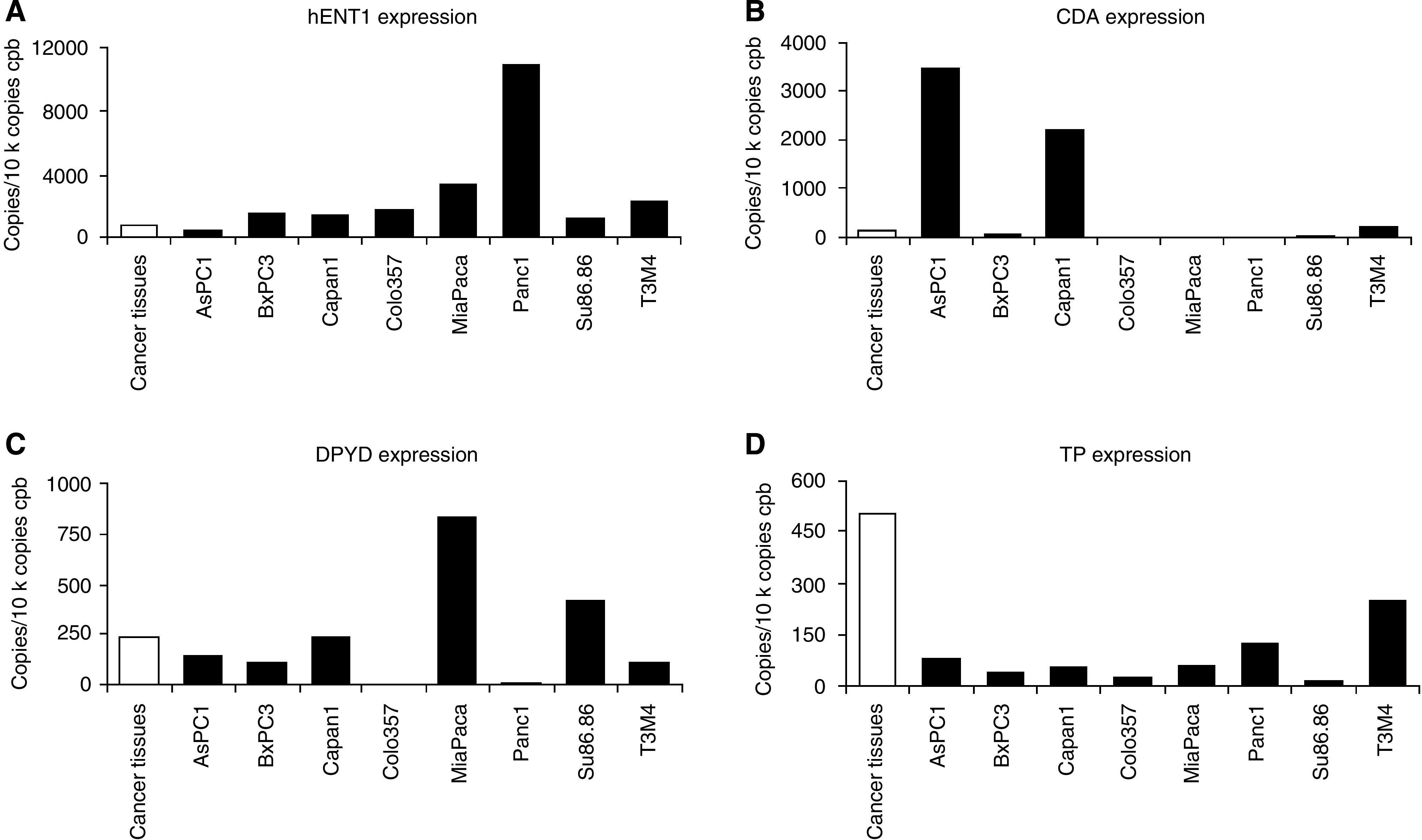
(**A**–**D**) Expression of human equilibrative nucleoside transporter-1 (hENT1) (**A**), cytidine deaminase (CDA) (**B**), dihydropyrimidine dehydrogenase (DPYD) (**C**) and thymidine phosphorylase (TP) (**D**) in eight pancreatic cancer cell lines (black bars) compared with the respective mean expression levels in pancreatic cancer patient tissues (white bars).

To compare expression profiles of primary isolated pancreatic cancer cells and established pancreatic cancer cell lines, gene expression levels of hENT1, CDA, DPYD and TP were analysed in all cell lines used before. The expression levels of these genes varied considerably between the tested cell lines. Some cell lines showed expression levels of individual genes comparable with the mean expression of the respective genes in the tested tumour specimens (Figure 4A–D; white bars show mean tumour tissue expression levels; black bars show expression levels in pancreatic cancer cell lines).

### Correlation analysis of chemosensitivity and gene expression in patients

When the size of a biopsy sample is insufficient to give enough cells for ATP-TCA, prediction of chemosensitivity using gene expression analysis by QRT–PCR may be an option. Thus, gene expression levels of hENT1, CDA, DCK, DPYD and TP were analysed for correlation with the IC_50_ levels obtained by ATP-TCA. As shown in Table 2, Spearman rho analysis revealed trends towards correlation of hENT1 with gemcitabine (*P*=0.099), of CDA with paclitaxel (*P*=0.066), of TP and gemcitabine or oxaliplatinum (*P*=0.068 and *P*=0.069, respectively) and of the TP/DPYD ratio and oxaliplatinum (*P*=0.058). Statistically significant correlations were observed for hENT1 and cisplatinum (*P*=0.049), for TP and gemcitabine+mitomycin C (*P*=0.047), for TP/DPYD and gemcitabine and cisplatinum (*P*=0.022 and *P*=0.025, respectively). To assess whether specific gene expressions can be used to predict *ex vivo* chemoresistance, we calculated the mean IC_50_ levels for all tested chemotherapeutic drugs and compared these with the expression levels of hENT1, CDA, DCK, DPYD and TP for correlation. This analysis demonstrated that hENT1 (directly) and TP expression (inversely) are related to the ‘mean’ chemosensitivity of pancreatic cancer cells (*P*=0.01 and *P*=0.014, respectively). The best predictor of chemosensitivity is the TP/DPYD ratio, which correlates highly significantly (*P*=0.001) with the mean IC_50_ levels of all tested chemotherapeutics.

### Correlation analysis of chemosensitivity and gene expression in cell lines

To assess whether the commonly used pancreatic cancer cell lines reflect the results obtained with primary isolated human pancreatic cancer cells, correlation analyses of the IC_50_ levels of these cells and their gene expression profiles (hENT1, CDA, DCK, DPYD and TP; Table 3) were performed. In contrast to the results from primary isolated cells, the most striking observation is a highly significant correlation of gemcitabine, gemcitabine+oxaliplatinum and gemcitabine+mitomycin C chemosensitivity with the expression levels of CDA (*P*=0.0003, *P*=0.01 and *P*=0.002, respectively) and a trend towards a relationship between CDA expression and the mean IC_50_ level of all tested drugs (*P*=0.057). Furthermore, a correlation was found between cisplatinum chemosensitivity and DPYD expression (*P*=0.004).

### *Ex vivo* chemosensitivity predicts time to relapse in gemcitabine-treated patients

**Figure 5:**
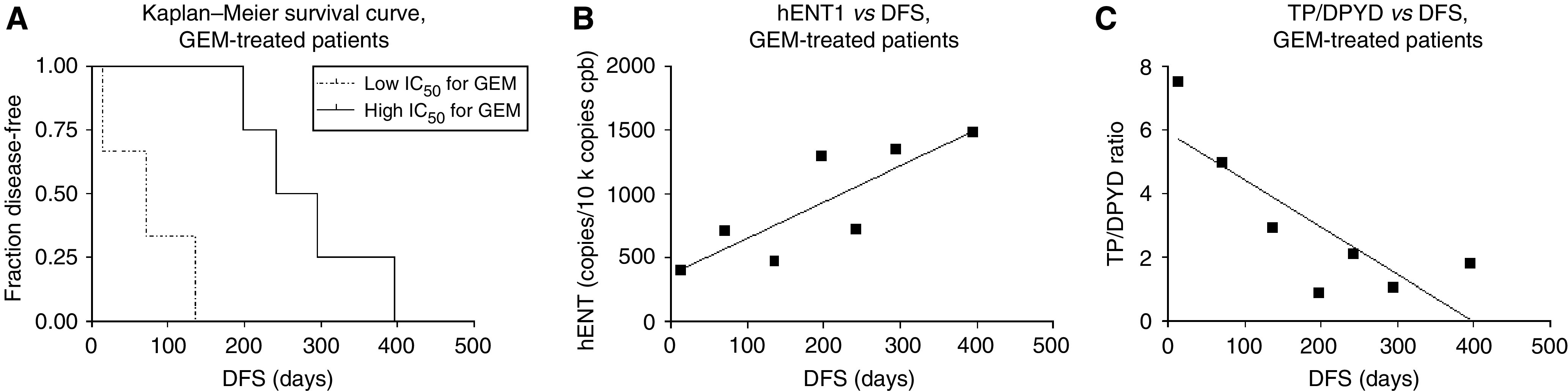
(**A**) Kaplan–Meier survival curve of gemcitabine-treated, ATP-TCA-tested patients (log-rank test: chemoresistant (dotted line) *vs* chemosensitive (continuous line) patients: *P*=0.01). (**B**) Correlation analysis of human equilibrative nucleoside transporter-1 (hENT1) expression *vs* time to relapse (DFS): Spearman *r*=0.9286, *P*=0.007; linear regression *F*=11.48, *P*=0.02. (**C**) Correlation analysis of thymidine phosphorylase (TP)/dihydropyrimidine dehydrogenase (DPYD) ratio *vs* time to relapse (DFS): Spearman rho *r*=−0.75, *P*=0.066; linear regression *F*=9.595, *P*=0.027.

Although only seven patients in this ATP-TCA-tested cohort received adjuvant first-line treatment with gemcitabine, time to relapse and chemosensitivity values were compared. In the group of patients whose primary cancer cells were resistant towards gemcitabine in the ATP-TCA, the median time to relapse was 71 days, whereas in the group of patients whose cancer cells exhibited sensitivity towards gemcitabine, the median time to relapse was 269 days (*P*=0.01; Figure 5A). Thus, *ex vivo* testing for gemcitabine chemosensitivity using the ATP-TCA may predict time to relapse in patients who are treated adjuvantly with gemcitabine (Figure 5A).

### Correlation analysis of gene expression profiles and disease-free survival in gemcitabine-treated patients

For small surgical specimens and biopsies with insufficient cell numbers for performance of the ATP-TCA, we tested whether molecular markers (ie, expression levels of hENT1, CDA, DCK, DPYD or TP as quantified by QRT–PCR) may be useful for predicting chemosensitivity/chemoresistance in the palliative setting. Expression levels of these genes were compared with the time to relapse of the seven patients who were treated with gemcitabine. These analyses showed that increased hENT1 expression is significantly related to the time to relapse (Spearman *r*=0.9286, *P*=0.007; linear regression *F*=11.48, *P*=0.02; Figure 5B). Furthermore, the TP/DPYD ratio is inversely related to time to relapse (Spearman rho *r*=−0.75, *P*=0.066; linear regression *F*=9.595, *P*=0.027; Figure 5C). Thus, these markers may be useful for predicting gemcitabine chemosensitivity for unresectable patients with tissue specimens obtainable only through a biopsy.

## Discussion

A central result of this study is that *ex vivo* chemosensitivity testing using the ATP-TCA is feasible in pancreatic cancer tissue specimens from resected patients and can be used for the potential planning of adjuvant treatment. Test results were obtained within a reasonable time of approximately 7 days, which allowed chemotherapy to be started immediately after the healing process was completed. Gene expression profiling of single genes using QRT–PCR could be performed in a standardised way and (at least partially) predicted the *in vitro* ATP-TCA response towards gemcitabine. Interestingly, the *in vitro* response of primary pancreatic cancer cells to various other nonstandard chemotherapeutics was also highly variable in the ATP-TCA.

Pancreatic cancer cells from various patients were very susceptible to paclitaxel, a drug exhibiting low toxicity compared with other cytostatic agents. This indicates that paclitaxel may be effective in pancreatic cancer when used selectively according to the individual chemosensitivity profiles. In contrast, the majority of isolated primary cancer cells were resistant to cisplatinum and oxaliplatinum as well as to mitomycin C. As these chemotherapeutics are usually associated with increased rates of toxicity (as compared with gemcitabine and 5-fluorouracil), the lack of *in vitro* cytotoxicity underlines the notion that these drugs should only be used in selected cases and should be excluded from the standard treatment regimens.

Although we could show that overall chemosensitivity of a set of eight pancreatic cancer cell lines was comparable to the chemosensitivity of isolated primary human pancreatic cancer cells, the actual sensitivity or resistance against a specific agent depends on the individual tumour/cell line. For *in vitro* chemoresistance testing of promising anticancer agents, it should be a prerequisite to use at least the whole set of pancreatic cancer cell lines instead of evaluating candidates with a single cell line.

Another important finding of our study is a correlation between *in vitro* chemosensitivity results and time to relapse in gemcitabine-treated patients. There was not only a trend but also a significant difference in chemotherapy response, with a much longer time to progress in patients with chemosensitive tumour cells. The short follow-up and the low number of patients precludes these results from being representative of a larger patient cohort. Therefore, large-scale trials will be necessary to define the clinical value of test-directed chemotherapy for pancreatic cancer.

Although chemosensitivity testing has been a controversial topic for a long time (Fruehauf and Alberts, 2005; Nagourney, 2005; Wieand, 2005) and randomised trials are definitely needed, test-directed chemosensitivity treatment could be offered to pancreatic cancer patients. The decision to be made in the adjuvant setting is not between a large number of cytotoxic drugs but rather between gemcitabine and 5-fluorouracil (Neoptolemos *et al*, 2004; Stocken *et al*, 2005; Oettle *et al*, 2007). Further studies such as ours might reveal other chemotherapeutic agents, which would be efficient in treatment of individual adenocarcinomas of the pancreas. In our opinion, and in line with a recent comment on *in vitro* chemosensitivity testing (Castro, 2005), test-directed chemotherapy is justified even in a smaller cohort of patients under randomised conditions.

Further, defining markers predictive of chemosensitivity is a prerequisite for inoperable patients for whom only marginal amounts of tissue are available. This might be possible by performing gene expression profiling using QRT–PCR or microarray analyses. In our study, there was a correlation between gemcitabine response and hENT1 expression, confirming what has already been shown in the literature in retrospective studies and *in vitro* experiments. However, the robustness of a single marker seems questionable, and thus further efforts are necessary to define a set of marker genes, which are really predictive of chemosensitivity and which are not just surrogates for survival. Therefore, controlled clinical trials using test-directed chemotherapies are urgently needed in the palliative situation. The markers that are found in such a patient cohort will then also be promising candidates for predicting the response towards adjuvant chemotherapy.

In conclusion, we have shown that *in vitro* chemosensitivity testing using the ATP-TCA test or expression analysis of the drug transporter hENT1 is feasible and may predict the response to gemcitabine chemotherapy in pancreatic cancer. A randomised controlled trial should be initiated to test the large-scale value of test-directed chemotherapy in the adjuvant and palliative treatment of pancreatic cancer.

## Figures and Tables

**Table 1:** Demographic characteristics of patients

**Characteristics**	**Number of patients**
Patients enrolled	24
Patients eligible	18
Men	11
Women	7
	
Median age (range)	56 (40–69) years
	
*T and N stages*	
T1, T2	0
T3	17
T4	1
N0	2
N1	16
	
*Grade*	
2	12
3	6

**Table 2:** Correlation analysis: mean IC_50_ levels of primary isolated pancreatic cancer cells *vs* gene expression

	**Gene expression**					
**CTx**	**hENT1**	**CDA**	**DCK**	**DPYD**	**TP**	**TP/DPYD**
5-fluorouracil	0.269	0.863	0.966	0.966	0.295	0.231
gemcitabine	***0.099***	0.774	0.91	0.684	***0.068***	***0.022***
Oxaliplatinum	0.221	0.296	0.88	0.81	***0.069***	***0.058***
Cisplatinum	**0.049**	0.191	0.82	0.459	0.056	**0.025**
Paclitaxel	0.425	***0.066***	0.465	0.564	0.641	0.754
Mitomycin C	0.405	0.800	0.621	0.94	0.232	0.191
Gemcitabine+oxaliplatinum	0.169	0.725	0.599	0.982	0.076	0.220
Gemcitabine+mitomycin C	0.144	0.594	0.681	0.935	**0.047**	0.169
Mean IC_50_ levels	**0.01**	0.823		0.381	**0.014**	**0.001**

CDA=cytidine deaminase; DPYD=dihydropyrimidine dehydrogenase; hENTI=human equilibrative nucleoside transporter-1; TP=thymidine phosphorylase.

Bold face: *P*-value significant (<0.05); italic face: *P*-value near significance level.

**Table 3:** Correlation analysis: mean IC_50_ levels of pancreatic cancer cell lines *vs* gene expression

	**Gene expression**				
**CTx**	**hENT1**	**CDA**	**DPYD**	**TP**	**TP/DPYD**
5-fluorouracil	0.432	0.223	0.489	0.39	0.282
Gemcitabine	0.424	**0.0003**	0.988	0.679	0.49
Oxaliplatinum	0.387	0.977	0.632	0.539	0.42
Cisplatinum	0.96	0.975	**0.004**	0.805	0.485
Paclitaxel	0.723	0.26	0.982	0.730	0.703
Mitomycin C	0.115	0.595	0.356	0.976	***0.104***
gemcitabine+oxaliplatinum	0.723	**0.01**	0.57	0.514	0.664
Gemcitabine+mitomycin C	0.769	**0.002**	0.893	0.72	0.776
					
Mean IC_50_ levels	0.939	**0.057**	0.597	0.875	0.955

CDA=cytidine deaminase; DPYD=dihydropyrimidine dehydrogenase; hENTI=human equilibrative nucleoside transporter-1; TP=thymidine phosphorylase.

Bold face: *P*-value significant (<0.05); italic face: *P*-value near significance level.
